# Prevalence of autism spectrum disorders and their relation to selected socio-demographic factors among children aged 18–30 months in northern Vietnam, 2017

**DOI:** 10.1186/s13033-019-0285-8

**Published:** 2019-04-29

**Authors:** Van Minh Hoang, Thi Vui Le, Thi Thuy Quynh Chu, Bich Ngoc Le, Minh Duc Duong, Ngoc Minh Thanh, Van Tac Pham, Harry Minas, Thi Thu Ha Bui

**Affiliations:** 1 Department of Health Economics and Finance, Hanoi University of Public Health, Hanoi, Vietnam; 2 Department of Demography-Reproductive Health, Hanoi University of Public Health, Hanoi, Vietnam; 3 Department of Biostatistics, Hanoi University of Public Health, Hanoi, Vietnam; 4 Department of Psychiatry, Vietnam National Hospital of Pediatrics, Hanoi, Vietnam; 5 Departments of Personnel and Organization, Vietnam Ministry of Health, Hanoi, Vietnam; 6 Global and Cultural Mental Health Unit, Center for Mental Health, Melbourne School of Population and Global Health, Melbourne, Australia

**Keywords:** Autism, Prevalence, Socio-demographic, Vietnam

## Abstract

**Background:**

Autism spectrum disorders are increasing worldwide and in Vietnam. This study reports the prevalence of autism spectrum disorders and examines their relation to selected socio-demographic factors among children aged 18 and 30 months in three northern cities/provinces in Vietnam, 2017.

**Methods:**

This was a cross-sectional study conducted among 17,277 children aged 18 and 30 months one city (Hanoi capital) and two provinces in northern Vietnam. The multi-stage sampling technique was applied in this study. We used M-CHAT to screen children with high risk of ASD. M-CHAT positive cases were diagnosed by pediatric neurologists from National Pediatrics Hospital using DSM-IV criteria. Descriptive and analytical statistics were performed.

**Results:**

The overall prevalence of ASD among children aged 18 and 30 months in the three studied sites was 0.752% (95% CI 0.629–0.893%). The odds of having ASD were statistically significant higher among (a) children living in urban area as compared to those from rural settings (OR = 2.7, 95% CI 1.73–4.21); (b) boys as compared to girls (OR = 4.04, 95% CI 2.57–6.35); and (c) children of mothers who worked as farmers as compared to children of mothers who were government staff (OR = 4.72, 95% CI 2.03–10.97).

**Conclusions:**

Our study revealed that the prevalence of ASD among children in Vietnam seems to be increasing. The significant correlates of ASD among the children were urban setting, male gender and mother’s occupation (farmer). Further and more in-depth studies on determinants of ASD are needed to provide insights into the problem.

## Introduction

Autism spectrum disorders (ASD) refer to a range of conditions characterized by some degree of impaired social behavior, communication and language, and a narrow range of interests and activities that are both unique to the individual and carried out repetitively [1]. ASD often impose significant emotional and economic burden on people with these disorders and their families [1, 2]. The impairments associated with ASD are present during the life-course [3] and are considered to have a substantial functional, social and financial impacts on affected individuals, their families and society.

Autism spectrum disorders prevalence appears to be increasing worldwide, with reported prevalence of about 0.5–1% [4]. In European countries, the prevalence of ASD, with an age range of birth to adulthood, varied from 0.019 to 7.2% [5]. The prevalence of ASD in the US in 2012 was 1.13% [6]. A recent systematic review reported the increasing trend in prevalence of ASD in South Asia, ranging from 0.09% in India to 1.07% in Sri Lanka [7]. ASD prevalence estimates were 0.118% in China in the last few years [8] and 2.64% in South Korea in 2011 [9].

In Vietnam, hospital statistics show that the number of cases of ASD admitted to the National Pediatrics Hospital increased more than threefold during the period 2000–2007 [10, 11] and fourfold during the period 2008–2010 [12]. A small-scale population-based study done by Giang et al. in 2012 in Thai Binh (a northern province of Vietnam) found the prevalence of ASD among children aged 18–24 months was 0.46% [13]. Another study conducted in Thai Nguyen in 2013 found that the prevalence of ASD among children aged 18–60 months was 0.52% [14]. In 2014, a screening program by Yen et al. [15] implemented among 94,186 children aged 18–60 months in three provinces/cities in the North of Vietnam found an ASD prevalence of 0.415%.

To provide information about epidemiologic aspects of ASD in Vietnam, this study investigated the prevalence of autism spectrum disorders and examined their relation to selected socio-demographic factors among children aged 18–30 months in three northern cities/provinces in Vietnam in 2017.

In this study, we used M-CHAT for screening ASD among the studied children as it has been widely used in other settings [16]. M-CHAT was proven to be an accessible, low-cost option for universal toddler screening [17]. The M-CHAT has also been validated internationally and estimated to have the positive predictive value (PPV) varying from 0.57 to 0.65 in low-risk samples [18–20]. M-CHAT has been translated into multiple languages [21] including Vietnamese. M-CHAT is currently being used as the main screening tool in clinical settings by physicians at the Vietnam National Pediatric Hospital with a positive predictive value (PPV) as high as 0.763 in 6583 children aged 18–24 months [13]. However, despite high PPV, sensitivity and specificity have not been clearly determined [22] and it is necessary to identify the percentage of false negatives [23]. We used DSM-IV for diagnosis of ASD because it has been previously applied in a variety of settings [7], including Vietnam [13]. In 2013, DSM-V was introduced and changed ASD diagnostic criteria, particularly eliminating diagnosis of a number of subtypes [24]. However, DSM-V was shown to be likely to decrease the number of individuals diagnosed with ASD [25–27].

## Methods

### Design

This was a cross-sectional study of population-representative samples of children in nine study sites (urban and rural districts) located in three cities/provinces in northern Vietnam.

### Research participants

Children aged 18 and 30 months in Vietnam who were living with their parents or caregivers at the time of research.

### Research sites

The research was implemented in one city (Hanoi, capital of Vietnam) and two provinces (Thai Binh province in the Red River Delta Region and Hoa Binh province in the Northern Midlands and Mountainous Region). These research sites were purposively selected as they have typical characteristics of the region in which they are located in terms of socio-economic conditions, population structure and health system.

### Sample size

The sample for each city/province was 5918 children. We estimated the sample size using the following formula:

$$n = z_{{\left( {1 - \alpha /2} \right)}}^{2} \frac{1 - p}{{\varepsilon^{2} p}}$$

In which, n was sample size at each study city/province; z _(1−α/2)_ was equal to 1.96 (confidence level of 95%); ɛ was relative precision (35%); p was anticipated population proportion of children with ASD (0.46% [13]); and the estimated refusal rate of 15%. The total sample size was 17,754 children.

### Sampling

Based on our experiences, we applied multistage sampling strategy. In the 1st stage, we selected a major city Hanoi and two provinces based on two socioeconomic regions in the northern Vietnam (as in the Decree No. 92/2006/ND-CP of 7 September 2006 on the establishment, approval and management of the general planning for socioeconomic development.) (as described in “Research sites” above). In the 2nd stage, we randomly selected one urban district and two rural districts in each city/province. A complete list of all children aged 18–30 months in the selected districts was compiled with the assistance of the District Health Center and Center of Population and Family Planning. In the 3rd stage, about 2000 children in each of the nine study districts were randomly selected from the lists of the children compiled in the 2nd stage.

### Screening and diagnosis

#### Screening phase

We used the 23 item Modified Checklist for Autism in Toddlers (M-CHAT) to screen children with high risk of ASD. M-CHAT is a screening tool for ASD which has been being used worldwide [22]. In Vietnam, M-CHAT was shown to have sensitivity of 74.4% and specificity of 99.9% in screening ASD [13]. During the research design phase, we reviewed and considered different autism screening tools, including M-CHAT and M-CHAT-R/F. After the careful consideration, we chose M-CHAT as the screening tool in our study for a number of reasons. M-CHAT had been translated and validated in the Vietnamese context [13]. M-CHAT was also used in some other previous studies in Vietnam. This tool has been also commonly used to screen autism among children in clinical settings in a number of hospitals in Vietnam. Moreover, owing to financial limitations, M-CHAT was more much appropriate for our study than M-CHAT-R/F that required follow-up after screening. The screenings were conducted at the children’s homes with the children’s parents.

#### Diagnostic confirmation phase

**Fig. 1:**
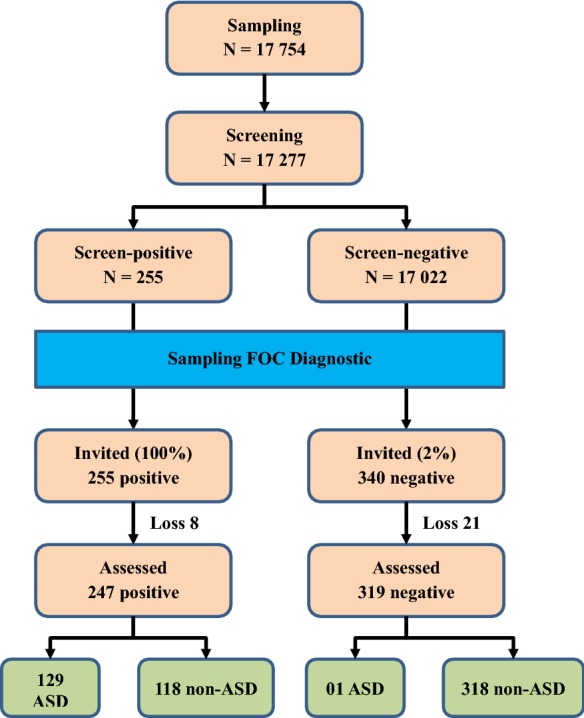
Screening and diagnostic process

All children of screen-positive group and 2% children of screen-negative group were invited to diagnostic confirmation assessment. Right after a screen-positive case was detected, the diagnosis was carried out at district health centers by eight doctors (04 pediatric neurologists and 04 psychologist) from National Hospital of Pediatrics using DSM-IV criteria. Each of screen-positive or screen-negative cases was diagnosed by two doctors (01 pediatric neurologists and 01 psychologist). These professionals have been performed the autism diagnosis and intervention among children for years at the hospital. The average length of time for the diagnosis was 35 min/case. The doctors were trained on DSM IV by the head of the Department of Psychiatry, National Hospital of Pediatrics who is pediatric psychologist. Figure 1 illustrates the process of ASD screening and diagnosis. There were 8 positive cases and 21 negative cases of loss to follow-up due to refusing to diagnose, moved to another province, the child was absent at the time of diagnosis, 03 children died. These professionals have been performed the autism diagnosis and intervention among children for years at the hospital.

### Study variables

The dependent variable was ASD, as initially identified by screening and subsequently diagnosed by pediatric neurologists, among the study participants. Independent variables included age and gender of the child, parents’ age, education and occupation, and the family’s economic status. Regarding parents, age was classified into three groups with (1) less than 29 years, (2) 29 to 39 years, and (3) 40 years and over. Education level was dichotomized into primary, secondary, high school, and university/college. Occupation status contained government and private sector staff, farmer, unstable job and other jobs. Family economy classified as poor when the annual household income was less than 1000 USD.

### Data collection

In each city/province, 46 local health workers (either medical doctor or nurse, midwives) were recruited and trained. 12 supervisors were assigned to supervise and monitor the survey data collectors throughout the process of data collection and to confirm positive screened cases at the household level. A team of pediatric neurologists, public health specialists and child development therapists provided 1 day of training on data collection to all the field data collection staff. The data collectors conducted face-to-face interviews with the mother/father or caregivers of the child using a structured questionnaire with three vital components including the children and their parents’ socio-demographic information, and 23 items of M-CHAT. The screening data were collected during July and September 2017. As noted above, M-CHAT was used as the primary screening tool for ASD and M-CHAT-positive cases were diagnosed by pediatric neurologists from National Pediatrics Hospital using DSM-IV criteria.

### Data analysis

Descriptive and analytical statistics were performed. Prevalence estimates were derived and stratified by socio-demographic variables. Binary logistic regression models were used to evaluate the odds of the dependent measure according to the demographic and socio-economic measures. An alpha level of 0.05 was used to determine statistical significance in all analyses. Data were analyzed using EPI Info 3.54 and Stata 14 software.

## Results

97.3% of children’s parents (17,277 out of 17,754) consented to participate in the study and completed the M-CHAT interviews. Of the 17,277 screened children, 255 (100%) screen-positive cases and 340 screen-negative cases (2%) were diagnosed by using DSM-IV criteria. There were 8 positive cases and 21 negative cases of loss to follow-up due to refusing to diagnose, moved to another province, the child was absent at the time of diagnosis, 02 children died. Of these 247 positive cases getting assessed, 129 were diagnosed with ASD. Of these 319 negative cases getting assessed, 01 was diagnosed with ASD.

Table 1 presents general characteristics of the study sample. Most of the study children (73.8%) lived in rural areas. There were more boys (53.6%) than girls (46.4%). More than half (58.7%) of the children were aged 24–30 months. The majority of the children’s mothers (67.4%) were aged less than 29 years. Most of the mothers (71.6%) had secondary school education or higher. Of the mothers 25.3% worked as farmers and 32.1% had unstable employment. Among the fathers 50.1% were less than 29 years and 45.9% were 29–39 years. Most of the fathers (70.3%) had completed secondary school education or higher. 22.2% of fathers worked as farmer and 43.3%, had unstable employment. Almost all the children’s households (91%) were classified as non-poor.

**Table 1:** General characteristics of the study participants

	Frequency	Percentage (%)
City/province		
Ha Noi	5501	31.8
Thai Binh	5883	34.1
Hoa Binh	5893	34.1
Area		
Rural	12,754	73.8
Urban	4523	26.2
Gender		
Girl	8009	46.4
Boy	9268	53.6
Age (months)		
18–23	7128	41.3
24–30	10,149	58.7
Mother age		
Less than 29	11,649	67.4
29 to 39	4061	23.5
40 and older	1567	9.1
Mother’s education		
Primary education	4908	28.4
Secondary education	5847	33.8
High school	3419	19.8
University/College	3103	18.0
Mother’s occupation		
Government staff	2816	16.3
Private sector staff	3787	21.92
Farmer	4378	25.34
Unstable job	5537	32.05
Others	759	4.39
Father’s age		
Less than 29	8647	50.1
29 to 39	7933	45.9
40 and older	697	4.0
Father’s education		
Primary education	5123	29.7
Secondary education	6345	36.7
High school	2756	16.0
University/College	3053	17.7
Father’s occupation		
Government staff	2421	14.0
Private sector staff	3037	17.6
Farmer	3827	22.2
Unstable job	7484	43.3
Others	508	2.9
Number of children in the family		
1	6044	35.0
2	8637	50.0
3	2596	15.0
Economic status		
Non-poor	15,725	91.0
Near poor/poor	1552	9.0
Total	17,277	100.0

The overall prevalence of ASD among children aged 18 and 30 months in the three cities/provinces was 0.752% (95% CI 0.629–0.893%). Table 2 presents prevalence of ASD among children aged 18 and 30 months by socio-demographic characteristics. The prevalence of ASD was significantly higher in urban (1.238%) than in rural areas (0.580%). The prevalence of ASD was significantly higher among children of mothers who worked as farmers (1.054%) than among children of mothers who were employed as government staff (0.497%). There were no statistically significant differences in the prevalence of ASD by other socio-demographic variables.

**Table 2:** Prevalence of ASD among children aged 18 and 30 months

	Count	Percentage (%)	Lower bound of 95% CI (%)	Upper bound of 95% CI (%)
City/province				
Ha Noi	46	0.836	0.613	1.114
Thai Binh	41	0.697	0.501	0.944
Hoa Binh	43	0.730	0.529	0.982
Area				
Rural	74	0.580	0.456	0.728
Urban	56	1.238	0.937	1.605
Gender				
Girl	23	0.287	0.182	0.431
Boy	107	1.155	0.947	1.393
Age (months)				
18–23	55	0.772	0.582	1.003
24–30	75	0.739	0.582	0.925
Mother age				
Less than 29	81	0.695	0.553	0.864
29 to 39	41	1.010	0.725	1.367
40 and older	8	0.511	0.221	1.003
Mother’s education				
Primary education	32	0.652	0.461	0.921
Secondary education	46	0.787	0.590	1.049
High school	32	0.936	0.663	1.321
University/College	20	0.645	0.416	0.997
Mother’s occupation				
Government staff	14	0.497	0.272	0.833
Private sector staff	30	0.792	0.535	1.129
Farmer	36	1.054	0.577	1.137
Unstable job	42	0.759	0.547	1.024
Others	8	0.822	0.456	2.066
Father’s age				
Less than 29	53	0.613	0.459	0.801
29 to 39	72	0.908	0.711	1.142
40 and older	5	0.717	0.233	1.666
Father’s education				
Primary education	31	0.605	0.426	0.859
Secondary education	50	0.788	0.598	1.038
High school	25	0.907	0.614	1.339
University/College	24	0.786	0.527	1.170
Father’s occupation				
Government staff	13	0.537	0.286	0.916
Private sector staff	27	0.889	0.587	1.291
Farmer	18	0.470	0.279	0.742
Unstable job	65	0.869	0.671	1.106
Others	7	1.378	0.556	2.818
Number of children in the family				
1	39	0.645	0.459	0.881
2	69	0.799	0.622	1.010
3	22	0.847	0.532	1.280
Economic status				
Non-poor	118	0.750	0.622	0.898
Near poor/poor	12	0.773	0.400	1.347
Total	130	0.752	0.629	0.893

Binary logistic regression analyses of correlates of ASD are presented in Table 3. The odds of having ASD were significantly higher among (a) children living in urban than in rural areas (OR = 2.7, 95% CI 1.73–4.21); (b) boys as compared to girls (OR = 4.04, 95% CI 2.57–6.35); and (c) children of mothers who worked as farmers as compared than in children of mothers who were employed as government staff (OR = 4.72, 95% CI 2.03–10.97).

**Table 3:** Binary logistic regression analyses of correlates of autism among children aged 18 and 30 months

	Odds ratio	Lower level of 95% CI	Upper level of 95% CI
Province			
Ha Noi	1		
Thai Binh	0.78	0.49	1.22
Hoa Binh	0.85	0.53	1.35
Area			
Rural	1		
Urban	2.70	1.73	4.21
Gender			
Girl	1		
Boy	4.04	2.57	6.35
Age (months)			
18–23	1		
24–30	1.00	0.70	1.41
Mother age			
Less than 29	1		
29 to 39	1.13	0.71	1.79
40 and older	0.72	0.33	1.58
Mother’s education			
Primary education	1		
Secondary education	1.07	0.62	1.85
High school	1.28	0.65	2.55
University/College	0.75	0.31	1.79
Mother’s occupation			
Government staff	1		
Private sector staff	1.60	0.79	3.22
Farmer	4.72	2.03	10.97
Unstable job	1.50	0.72	3.12
Others	1.84	0.71	4.79
Father’s age			
Less than 29	1		
29 to 39	1.24	0.79	1.95
40 and older	0.89	0.30	2.62
Father’s education			
Primary education	1		
Secondary education	1.10	0.64	1.89
High school	1.29	0.64	2.61
University/College	1.33	0.57	3.11
Father’s occupation			
Government staff	1		
Private sector staff	1.73	0.83	3.60
Farmer	0.64	0.25	1.65
Unstable job	1.85	0.87	3.92
Others	2.71	0.92	8.01
Number of children in the family			
1	1		
2	1.14	0.75	1.74
3	1.17	0.63	2.19
Economic status			
Non-poor	1		
Near poor/poor	1.06	0.56	2.02

Table 4 shows the results of binary logistic regression analyses of correlates of autism among children aged 18 and 30 months with an interaction term of living area and gender of the child. After controlling for other variables in the model, boys living in rural areas had 3.13 times higher odds of having ASD than girls living in rural areas, and the difference was statistically significant (OR = 3.13, 95% CI 1.79–5.45). Boys living in urban areas had 9.46 times higher odds of having ASD than girls living in rural areas, and the difference was statistically significant (OR = 9.46, 95% CI 5.03–17.80). Children of mothers who worked as farmers had 4.71 times higher odds of having ASD than children of mothers who were government staff (OR = 4.71, 95% CI 2.03–10.94).

**Table 4:** Binary logistic regression analyses of correlates of autism among children aged 18 and 30 months with interaction term

	Odds ratio	Lower level of 95% CI	Upper level of 95% CI
Province			
Ha Noi	1		
Thai Binh	0.78	0.49	1.22
Hoa Binh	0.85	0.53	1.35
Gender * Area			
Girl * Rural	1		
Boy * Rural	3.13	1.79	5.45
Girl * Urban	1.54	0.61	3.92
Boy * Urban	9.46	5.03	17.80
Age (months)			
18–23	1		
24–30	1.00	0.70	1.42
Mother age			
Less than 29	1		
29 to 39	1.13	0.71	1.79
40 and older	0.72	0.33	1.57
Mother’s education			
Primary education	1		
Secondary education	1.07	0.62	1.85
High school	1.28	0.65	2.55
University/College	0.75	0.31	1.78
Mother’s occupation			
Government staff	1		
Private sector staff	1.60	0.79	3.23
Farmer	4.71	2.03	10.94
Unstable job	1.50	0.72	3.11
Others	1.82	0.70	4.74
Father’s age			
Less than 29	1		
29 to 39	1.24	0.79	1.94
40 and older	0.89	0.30	2.61
Father’s education			
Primary education	1		
Secondary education	1.10	0.64	1.89
High school	1.29	0.63	2.60
University/College	1.34	0.58	3.13
Father’s occupation			
Government staff	1		
Private sector staff	1.73	0.83	3.61
Farmer	0.64	0.25	1.66
Unstable job	1.86	0.87	3.94
Others	2.74	0.93	8.08
Number of children in the family			
1	1		
2	1.14	0.75	1.74
3	1.17	0.63	2.19
Economic status			
Non-poor	1		
Near poor/poor	1.06	0.56	2.00

## Discussion

Our study is one of few population-based studies of prevalence of ASD in children in Vietnam. Our findings show that the prevalence of ASD among children aged 18–30 months in northern Vietnam was 0.752% or 75.2 per 10,000 children. This is higher than figures reported by previous surveys in Vietnam (i.e. 0.46% [11] in 2007, 0.416–0.52% in 2013–2014 [13–15]) suggesting that ASD prevalence among the children in Vietnam may be increasing, consistent with the global trend of ASD currently.

The prevalence of ASD among the children found in this study is quite similar to the average prevalence of ASD in the world (0.76%) [28]. The prevalence of ASD among the children in Vietnam is lower than that of high-income countries (e.g. 2.41% among children and adolescents in the USA in 2014–2016 [29], 1% in Finland and Sweden and 1.5% in Denmark in 2011 [30]) as well as in Asia (e.g. 1.8% in children in Japan in 2008 [31], and 2.6% in 7- to 12-year-old children in South Korea [9]). The prevalence of ASD among children in Vietnam found in this study is similar to the figure of 0.9% in India in 2015 [32].

Odds of ASD for boys were higher than for girls. This is consistent with previous findings from other countries [33–37]. The discussion on gender also might need to be reviewed, as it is complex and is still not well investigated internationally.

While this may be so in older children it is unlikely to be a relevant consideration in children aged 18–30 months. It has also been suggested that sex chromosomal genes, and/or sex hormones, can lower the risk of ASD in girls [38].

Our study revealed that the prevalence of ASD was higher among children living in urban environments. This is concordant with findings from other studies in the world [39, 40]. Increasing degree of urbanization was associated with higher risk of ASD [41, 42]. It suggests that environment-related factors might contribute to this disparity. The exposure to hazardous air pollutants during pregnancy and early childhood may have potential association with increased risk for ASD [43–45] while hazardous air pollutants usually concentrate in urban environments. Hygiene practices commonly in urban areas lowered microbial exposure in pregnancy and neonatal life that affects the risk for ASD [46].

Our study also showed higher odds of ASD among children whose mothers worked as farmers. This finding suggests a possible relationship between ASD and farming practice, especially the common use of pesticides in Vietnam. The amount of chemicals used in agriculture has kept increasing during the period 2002–2013 in Vietnam [47]. Several international studies have shown the association between elevated ASD risk and exposure to agricultural chemicals [48–50]. The impact of chemicals on ASD should be further investigated in countries, such as Vietnam, where pesticide use is insufficiently well controlled.

Because autism is an uncommon childhood disorder a possible limitation of the study is that a larger sample size may be required to determine with greater confidence the prevalence of autism in very young children.

## Conclusion

This study found a prevalence of ASD among very young children that is higher than reported from previous studies in Vietnam, suggesting the possibility that prevalence of ASD may be increasing in Vietnam, as has been suggested by studies in other countries. The significant correlates of ASD among the children were urban setting, male gender and mother’s occupation (farmer). There is a need to study the underlying specific risks associated with male gender, living in urban settings and having mother who worked as farmer in order to develop possible preventative strategies. Regardless of the underlying causes of ASD, there is clearly also a need to develop more effective and more widely available early screening/detection, diagnosis and interventions programs for ASD in Vietnam. This will require attention and investment in the areas of policy-making and implementation, strengthening of relevant human resource capabilities in health and social affairs agencies and in schools, and in development of service programs that are effective and equitably available in the diverse regions of Vietnam.

## Abbreviations

ASD: autism spectrum disorders
DSM-IV: Diagnostic and Statistical Manual of mental disorders fourth edition
M-CHAT: The Modified Checklist for Autism in Toddlers
PPV: positive predictive value
